# Safe electrophysiologic profile of dexmedetomidine in different experimental arrhythmia models

**DOI:** 10.1038/s41598-021-03364-y

**Published:** 2021-12-14

**Authors:** Christian Ellermann, Jonas Brandt, Julian Wolfes, Kevin Willy, Felix K. Wegner, Patrick Leitz, Philipp S. Lange, Florian Reinke, Lars Eckardt, Gerrit Frommeyer

**Affiliations:** Department of Cardiology II (Electrophysiology), University Hospital Münster, Albert-Schweitzer-Campus 1, 48149 Münster, Germany

**Keywords:** Cardiology, Preclinical research

## Abstract

Previous studies suggest an impact of dexmedetomidine on cardiac electrophysiology. However, experimental data is sparse. Therefore, purpose of this study was to investigate the influence of dexmedetomidine on different experimental models of proarrhythmia. 50 rabbit hearts were explanted and retrogradely perfused. The first group (n = 12) was treated with dexmedetomidine in ascending concentrations (3, 5 and 10 µM). Dexmedetomidine did not substantially alter action potential duration (APD) but reduced spatial dispersion of repolarization (SDR) and rendered the action potentials rectangular, resulting in no proarrhythmia. In further 12 hearts, erythromycin (300 µM) was administered to simulate long-QT-syndrome-2 (LQT2). Additional treatment with dexmedetomidine reduced SDR, thereby suppressing torsade de pointes. In the third group (n = 14), 0.5 µM veratridine was added to reduce the repolarization reserve. Further administration of dexmedetomidine did not influence APD, SDR or the occurrence of arrhythmias. In the last group (n = 12), a combination of acetylcholine (1 µM) and isoproterenol (1 µM) was used to facilitate atrial fibrillation. Additional treatment with dexmedetomidine prolonged the atrial APD but did not reduce AF episodes. In this study, dexmedetomidine did not significantly alter cardiac repolarization duration and was not proarrhythmic in different models of ventricular and atrial arrhythmias. Of note, dexmedetomidine might be antiarrhythmic in acquired LQT2 by reducing SDR.

## Introduction

Dexmedetomidine is a selective α_2_-agonist increasingly used for sedation in intensive care medicine and anesthesia^1^. Sympatholytic effects of dexmedetomidine lead to sedation, analgesia, hypotension and reduce circulating plasma catecholamines^2^. Clinical data concerning its effect on cardiac repolarization is controversial: while most studies report a significant QT interval abbreviation after administration of dexmedetomidine^3–5^, other studies and case reports suggest a lengthening of QT intervals after dexmedetomidine treatment^6,7^. However, the sole measurement of the QT interval may be insufficient to determine the drug-induced proarrhythmic risk^8^. Other parameters such as transmural dispersion of repolarization or action potential shape need to be considered. In clinical studies, the duration from the peak to the end of the T wave of the ECG (T_peak_-T_end_ interval) is often regarded as surrogate for the transmural dispersion of repolarization. Some clinical studies report on a reduction of the T_peak_–T_end_ interval^9^ after dexmedetomidine treatment while others did not detect any significant effects^5^. However, it is worthy of note that an experimental study did not observe a significant correlation of the T_peak_–T_end_ interval with the transmural but rather with the total dispersion of repolarization^10^.

Plenty of clinical studies suggest an impact of dexmedetomidine on the occurrence of arrhythmias. A recent meta-analysis summarized the effects of dexmedetomidine in patients undergoing cardiac surgery and suggested a beneficial impact of perioperative administration of dexmedetomidine regarding the incidence of postoperative ventricular tachycardia and atrial fibrillation^11^. These findings are supported by an experimental study that found protective effects of dexmedetomidine against myocardial ischemia–reperfusion injury, resulting in less reperfusion-induced ventricular arrhythmias^12^. Of note, another in vivo study employing rabbit hearts found beneficial effects in the setting of acquired long QT syndrome^13^.

Dexmedetomidine exerts direct effects on different ion currents: Dexmedetomidine induces a concentration-dependent inhibition of the cardiac sodium channel Nav1.5 in vitro and inhibits the persistent sodium current induced by veratridine^14^. In addition, dexmedetomidine inhibits the amplitude of the calcium current independent of the α1- or α2-adrenoceptor, and the imidazoline receptor^15^. Of note, dexmedetomidine does not affect the potassium currents I_K1_ and I_Kr_ or the pacemaker current I_f_^15^ but influences the ATP-sensitive potassium current I_K,ATP_^16^.

However, experimental data investigating electrophysiologic mechanisms is sparse. Therefore, purpose of this study was to investigate the influence of dexmedetomidine on ventricular and atrial electrophysiology and the susceptibility to arrhythmias in a sensitive whole-heart model.

## Methods

All experimental protocols were approved by the local animal care committee (Landesamt für Natur, Umwelt und Verbraucherschutz Nordrhein-Westfalen, Germany) and were carried out in accordance with the ARRIVE guidelines and the Guide for the Care and Use of Laboratory Animals published by the US National Institutes of Health (NIH Publication No. 852-3, revised 1996). In this study, hearts were not randomized since they served as their own control.

**Figure 1:**
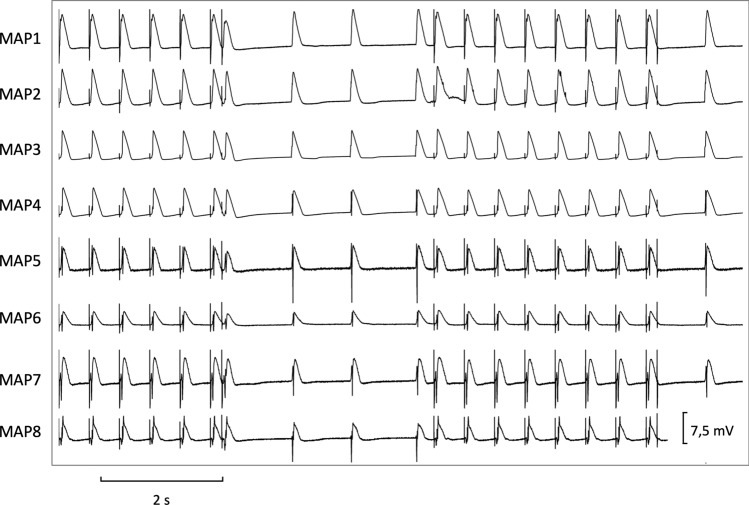
Determination of effective refractory periods at a basic cycle length of 500 ms (MAP = monophasic action potential).

**Figure 2:**
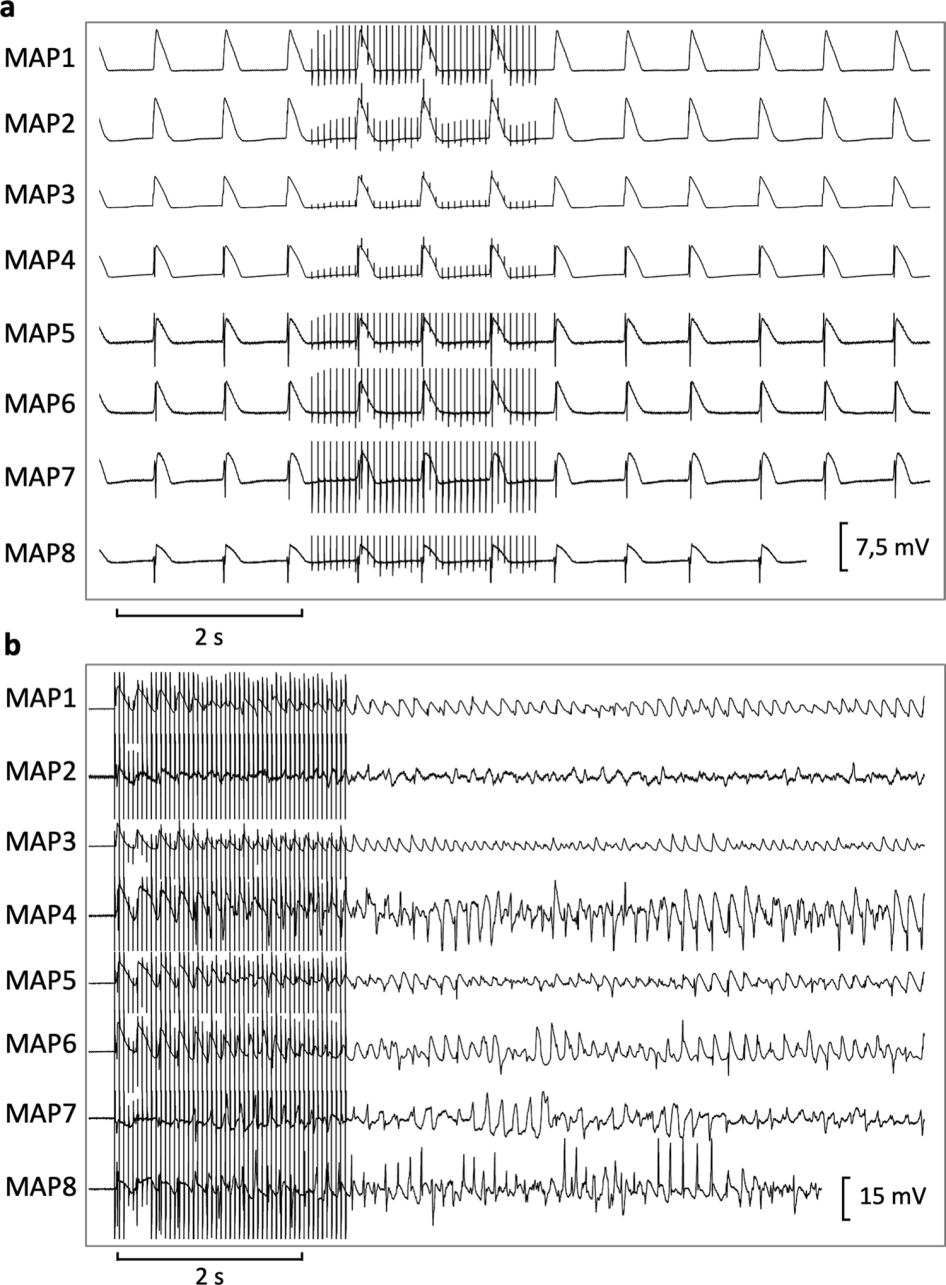
Trains of burst pacing without (**a**) and with (**b**) induction of ventricular fibrillation under baseline conditions (MAP = monophasic action potential).

The experimental Langendorff setup has been described earlier by our group^17,18^. In brief, 50 New Zealand White rabbit hearts were excised, attached to a Langendorff apparatus and retrogradely perfused. Spontaneously beating hearts were mechanically AV-node ablated by compressing the interatrial septum with surgical tweezers. Hearts were perfused with a warmed and oxygenated (95% O_2_, 5% CO_2_) modified Krebs–Henseleit buffer with a pH of 7.4 (NaCl 118 mM, NaHCO_3_ 24.88 mM, D-glucose 5.55 mM, KCl 4.70 mM, Na-pyruvate 2 mM, CaCl_2_ 1.80 mM, KH_2_PO_4_ 1.18 mM, MgSO_4_ 0.83 mM) at a constant flow (52 mL/h) with a pressure around 90 mmHg. Eight specifically designed catheters were placed endo- and epicardially, thereby recording monophasic action potentials. Simultaneously, a pseudo 12 lead ECG was recorded. Thereafter, the pacing protocol was started:

Firstly, hearts were stimulated at seven different cycle lengths (900–300 ms), thereby recording cycle-lengths dependent monophasic action potentials and QT intervals. Thereafter, effective refractory periods were assessed by delivering a short-coupled extrastimulus after a train of seven stimuli at each cycle length (900–300 ms, Fig. 1). Premature extrastimuli (S_2_ and S_3_) and burst stimulations (Fig. 2) were employed to test ventricular vulnerability. Afterwards, AV-blocked bradycardic hearts were perfused with a hypokalemic (1.5 mM) solution to trigger early afterdepolarizations and torsade de pointes. For examination of atrial electrophysiology, two catheters were clamped to both atria to record atrial monophasic action potentials. Atrial burst pacing manoeuvers were performed to assess atrial vulnerability.

Action potential duration at 90% of repolarization (APD_90_) was measured between the fastest upstroke and 90% of repolarization. Spatial dispersion of repolarization was determined by the difference of the maximum and the minimum of the eight simultaneously recorded monophasic action potentials. The ratio of APD_90_/APD_50_ was calculated to determine the action potential shape.

50 hearts were allocated to four groups. The first group (n = 12) was treated with dexmedetomidine in rising concentrations (3, 5 & 10 µM) after generating baseline data. To abridge the experimental protocol, effective refractory periods were solely determined at a basic cycle length of 500 ms in this group. The second group (n = 12) was perfused with 300 µM erythromycin to block I_Kr_ and thereby simulate long QT syndrome-2 (LQT2). In the third group (n = 14), 0.5 µM veratridine was administered, thus inhibiting sodium channel inactivation and consequently reducing the repolarization reserve^19^. Further 12 hearts were treated with a combination of acetylcholine (1 µM) and isoproterenol (1 µM) to facilitate atrial fibrillation (AF) and drug effects on atrial electrophysiology were investigated. In the latter three groups, dexmedetomidine (3 µM) was administered additionally to determine the influence of dexmedetomidine in acquired long QT syndrome and atrial fibrillation, respectively. Hearts were equilibrated at the new concentration for 15 min before the pacing protocol was started.

All electrolytes and drugs were acquired from Sigma Aldrich (Steinheim, Germany). Except from veratridine which was dissolved in dimethyl sulfoxide, all drugs were dissolved in deionized water. Dexmedetomidine, erythromycin, veratridine, acetylcholine and isoproterenol were administered separately employing syringe pumps via lines which were connected to the Langendorff-perfusion system.

### Statistics

Electrograms and action potentials were recorded on a multi-channel recorder and digitalized at a rate of 1 kHz with a 12-bit resolution. Variables are shown as mean ± standard deviation. Statistical analyses were performed employing SPSS Statistics for Windows (version 24.0). Drug effects on APD_90_, QT interval, spatial dispersion of repolarization and effective refractory periods were analysed employing a mixed model ANOVA. P values < 0.05 were considered to be statistically significant.

## Results

### Effects of dexmedetomidine on ventricular repolarization

**Figure 3:**
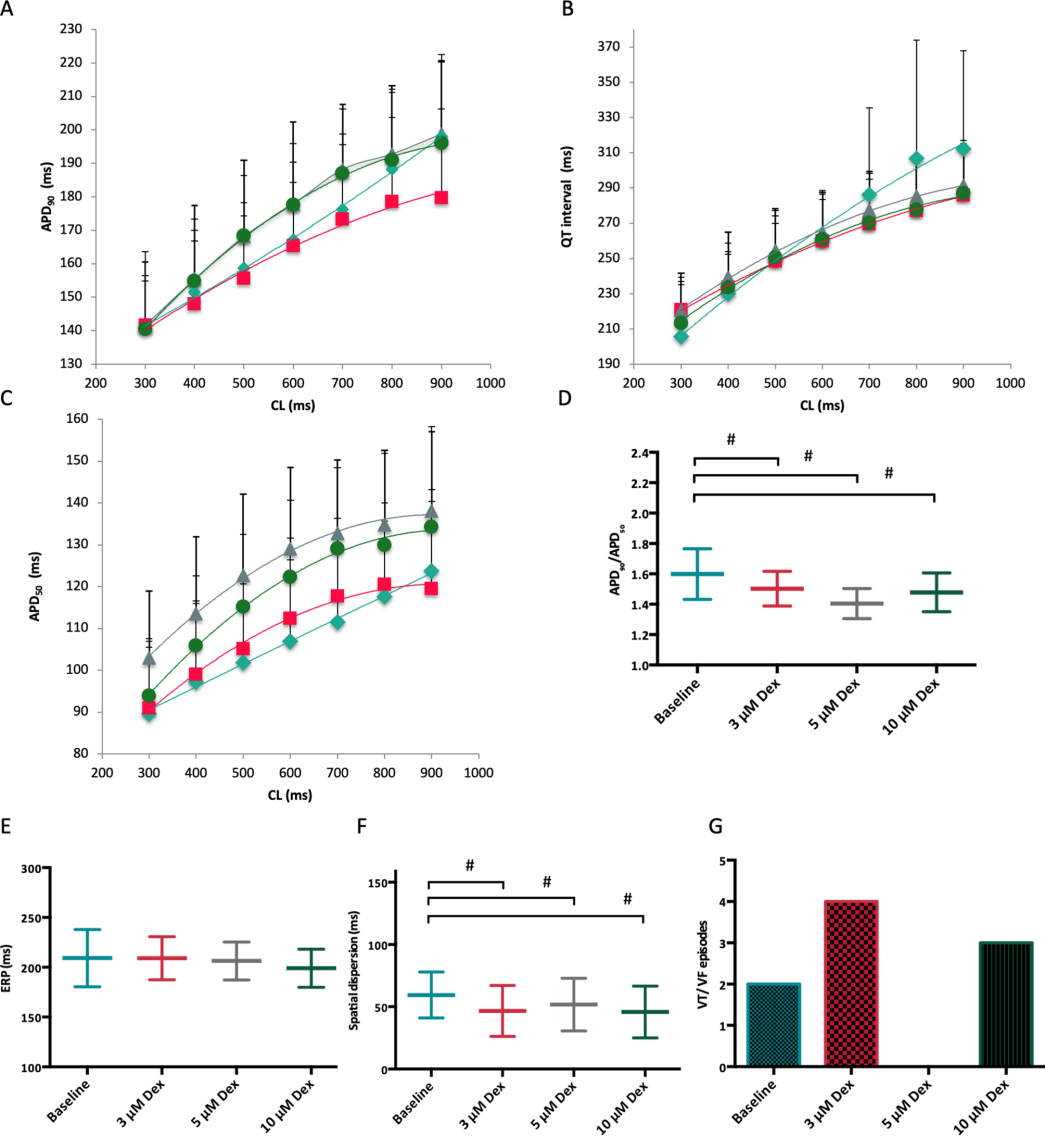
(**A**,**B**,**C**) Cycle-length dependent action potential durations at 90% of repolarization (APD_90_), QT interval and action potentials at 50% of repolarization (APD_50_) under baseline conditions (◆) and after treatment with 3 µM (■), 5 µM (▲) or 10 µM (●) dexmedetomidine (Dex). (**D**) Ratio of action potential duration at 90% and 50% of repolarization (APD_90_/APD_50_). (**E**) Concentration-dependent effect of dexmedetomidine on spatial effective refractory periods (ERP). (**F**) Impact of dexmedetomidine on spatial dispersion of repolarization. (**G**) Number of ventricular tachycardia (VT)/ fibrillation (VF) induced by programmed ventricular stimulation (# = p < 0.05 compared to baseline conditions).

Dexmedetomidine did not substantially alter QT interval or APD_90_ (Fig. 3A,B). Effective refractory periods remained unchanged after administration of dexmedetomidine (baseline: 209 ± 29 ms; 3 µM: 209 ± 22 ms, p = ns; 5 µM: 206 ± 19 ms, p = ns; 10 µM: 199 ± 19, p = ns). Spatial dispersion of repolarization was significantly reduced in the presence of dexmedetomidine (baseline: 59 ± 18 ms; 3 µM: 47 ± 19 ms, p < 0.01; 5 µM: 52 ± 22 ms, p < 0.01; 10 µM: 46 ± 20, p < 0.01). The ratio of APD_90_/APD_50_ was significantly reduced after administration of dexmedetomidine (baseline: 1.6 ± 0.17; 3 µM: 1.5 ± 0.11 ms, p < 0.01; 5 µM: 1.4 ± 0.1 ms, p < 0.01; 10 µM: 1.48 ± 0.13, p < 0.01), indicating a rectangulation of action potentials after dexmedetomidine treatment.

The occurrence of ventricular arrhythmias induced by programmed ventricular stimulation (Fig. 3) was not significantly amplified (baseline: 2 episodes; 3 µM: 4 episodes, p = ns; 5 µM: 0 episodes, p = ns; 10 µM: 3 episodes, p = ns). No torsade de pointes episodes were observed at any concentration in bradycardic hearts under hypokalemic conditions.

### LQT2 group

**Figure 4:**
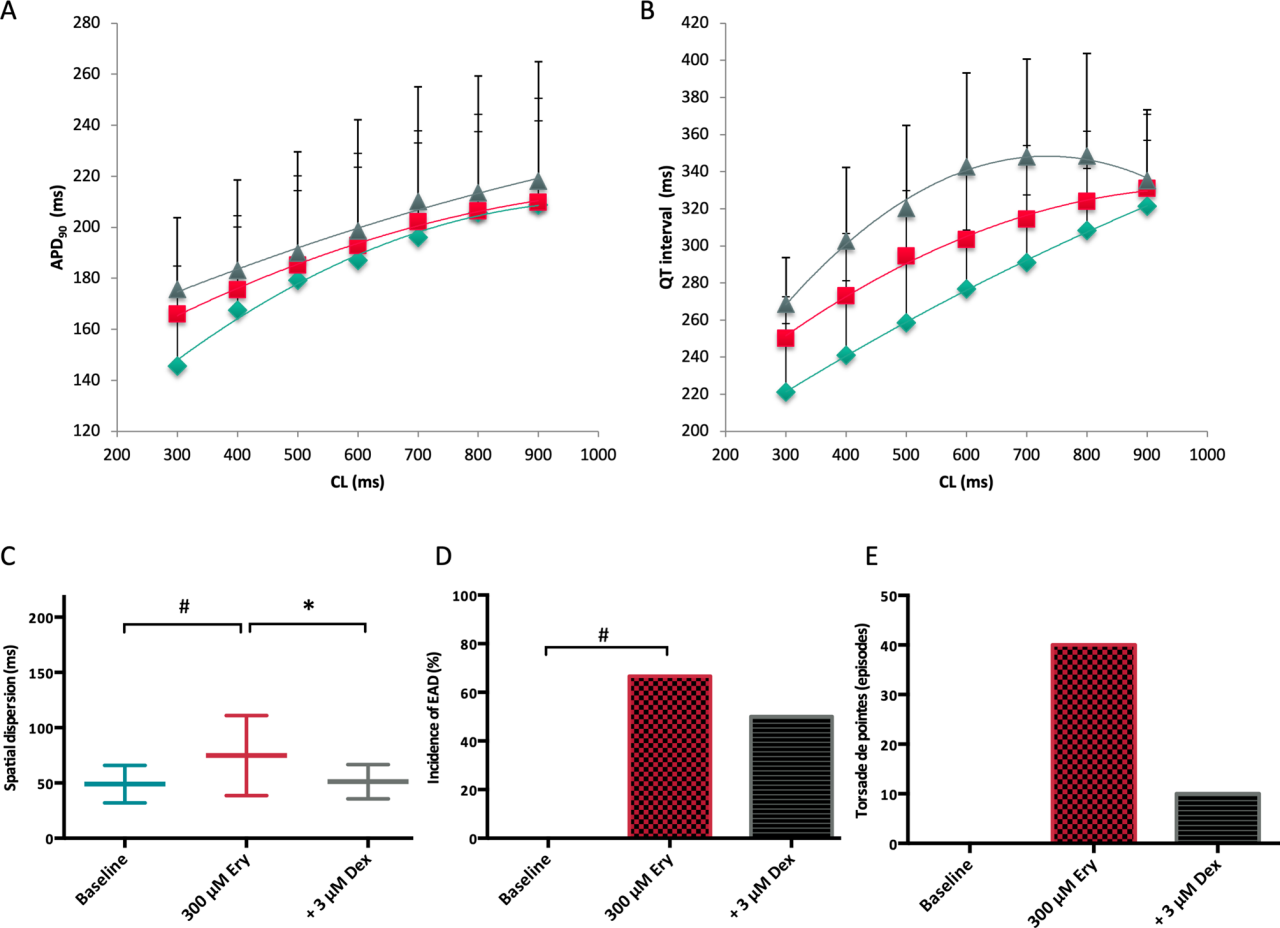
(**A**,**B**) Cycle-length dependent action potential durations (APD_90_) and QT interval under baseline conditions (◆), after infusion of 300 µM erythromycin (■) and after additional treatment with 3 µM dexmedetomidine (▲). (**C**) Impact of erythromycin (Ery) and dexmedetomidine (Dex) on spatial dispersion of repolarization. (**D**) Occurrence of early afterdepolarizations (EAD) after administration of erythromycin and after additional treatment with dexmedetomidine. (**E**) Incidence of torsade de pointes under baseline conditions, with erythromycin and with the combination of erythromycin and dexmedetomidine (# = p < 0.05 compared to baseline conditions; * = p < 0.05 compared to sole erythromycin infusion).

Administration of erythromycin significantly prolonged QT interval from 274 ± 36 ms to 299 ± 35 ms (p < 0.01) and APD_90_ from 184 ± 39 ms to 191 ± 28 ms (p < 0.05; Fig. 4). Simultaneously, spatial dispersion of repolarization was substantially amplified from 49 ± 16 ms to 75 ± 37 ms (p < 0.01). Effective refractory periods were prolonged in the presence of erythromycin (from 238 ± 42 ms to 261 ± 39 ms, p < 0.01). Further treatment with dexmedetomidine prolonged cardiac repolarization (QT interval: to 324 ± 44 ms, p < 0.01; APD_90_ to 198 ± 41 ms, p = 0.09) and reduced spatial dispersion of repolarization (to 51 ± 14 ms, p < 0.01). Effective refractory periods were prolonged after dexmedetomidine treatment (to 274 ± 46 ms, p < 0.01).

**Figure 5:**
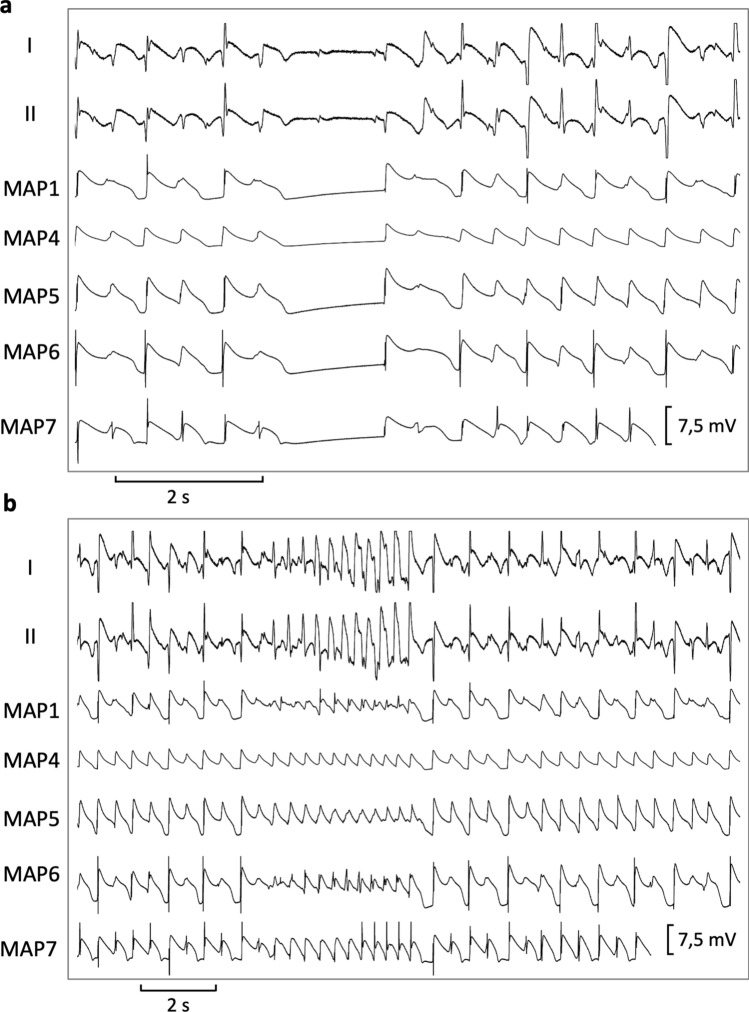
(A) Representative example of early afterdepolarizations (**a**) and torsade de pointes (**b**) induced by erythromycin (MAP = monophasic action potential).

No early afterdepolarization occurred under baseline conditions. Early afterdepolarizations were observed in 8 of 12 hearts with erythromycin (p < 0.01; Fig. 5) and in 6 of 12 hearts (p = ns) after additional treatment with dexmedetomidine. Erythromycin led to more torsade de pointes compared to baseline conditions (40 vs. 0 episodes, p = 0.06). Dexmedetomidine suppressed torsade de pointes substantially (10 episodes, p = 0.06 compared to erythromycin).

### Veratridine group

**Figure 6:**
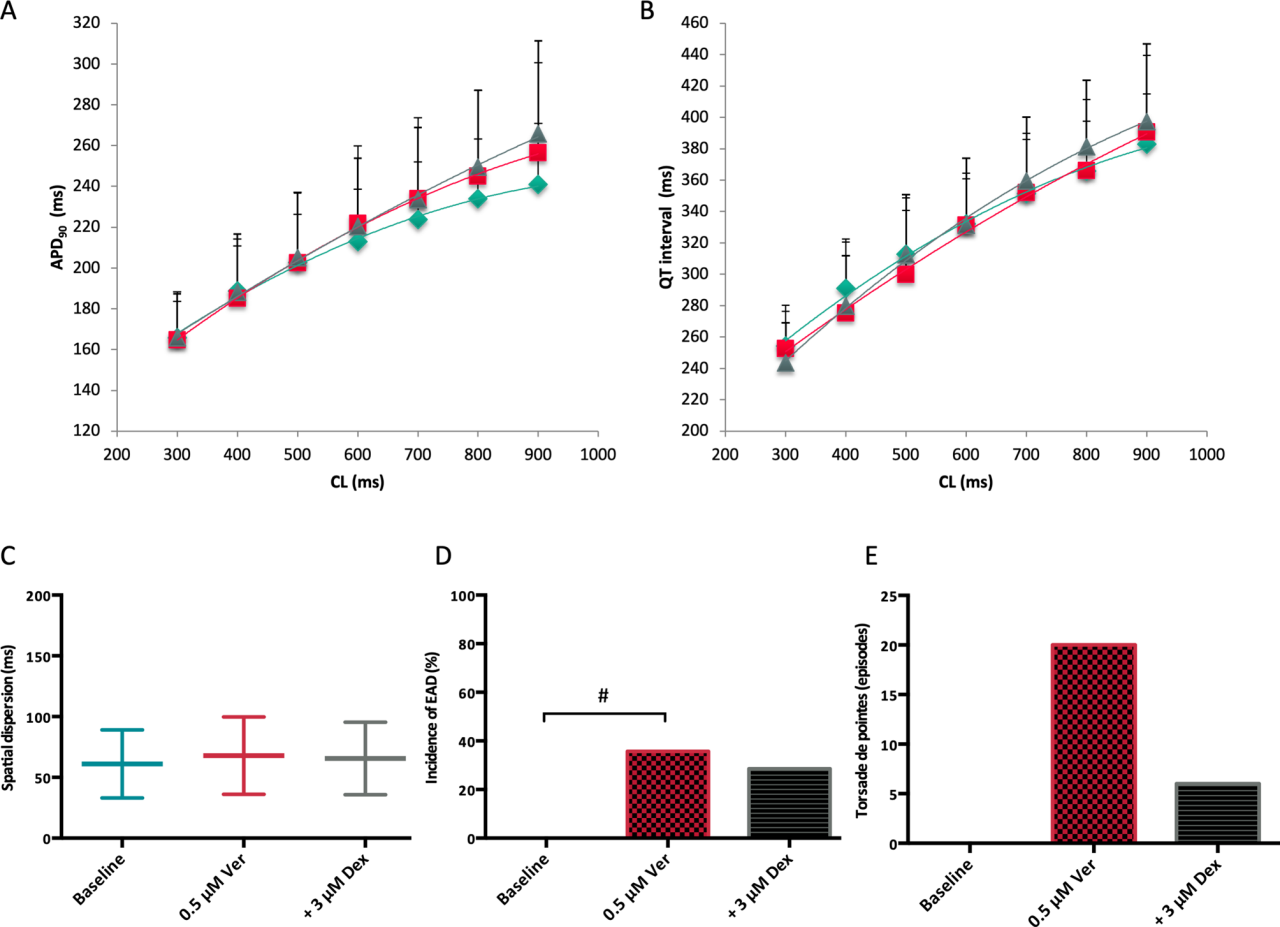
(**A**,**B**) Cycle-length dependent action potential durations (APD_90_) and QT interval under baseline conditions (◆), after infusion of 0.5 µM veratridine (■) and after additional treatment with 3 µM dexmedetomidine (▲). (**C**) Impact of veratridine (Ver) and dexmedetomidine (Dex) on spatial dispersion of repolarization. (**D**) Occurrence of early afterdepolarizations (EAD) after administration of veratridine and after additional treatment with dexmedetomidine. (**E**) Incidence of torsade de pointes under baseline conditions, with veratridine and with the combination of veratridine and dexmedetomidine (# = p < 0.05 compared to baseline conditions).

Veratridine slightly altered cardiac repolarization (QT interval: baseline: 327 ± 30 ms; veratridine: 324 ± 41 ms, p = ns; APD_90_: baseline: 210 ± 25 ms; veratridine: 216 ± 36, p = ns; Fig. 6) and spatial dispersion of cardiac repolarization (baseline: 61 ± 26 ms; veratridine: 68 ± 30, p = ns). Refractory periods remained unchanged in the presence of veratridine (baseline: 282 ± 40 ms; veratridine: 290 ± 50, p = ns). After additional treatment with dexmedetomidine, QT interval (to 329 ± 39, p = ns), APD_90_ (to 218 ± 33, p = ns) and spatial dispersion of repolarization (to 66 ± 29, p = ns) were not further amplified. Effective refractory periods were reduced in the presence of dexmedetomidine (to 283 ± 49, p < 0.05).

No early afterdepolarizations or torsade de pointes episodes occurred under baseline conditions. With veratridine, early afterdepolarizations occurred in 5 of 14 hearts (p < 0.05 compared to baseline) and 20 episodes of torsade de pointes (p = 0.1) were observed. There was a trend towards less triggered activity after administration of dexmedetomidine (early afterdepolarizations in 4 of 14 hearts, p = ns; 6 episodes of torsade de pointes, p = ns).

### AF group

**Figure 7:**
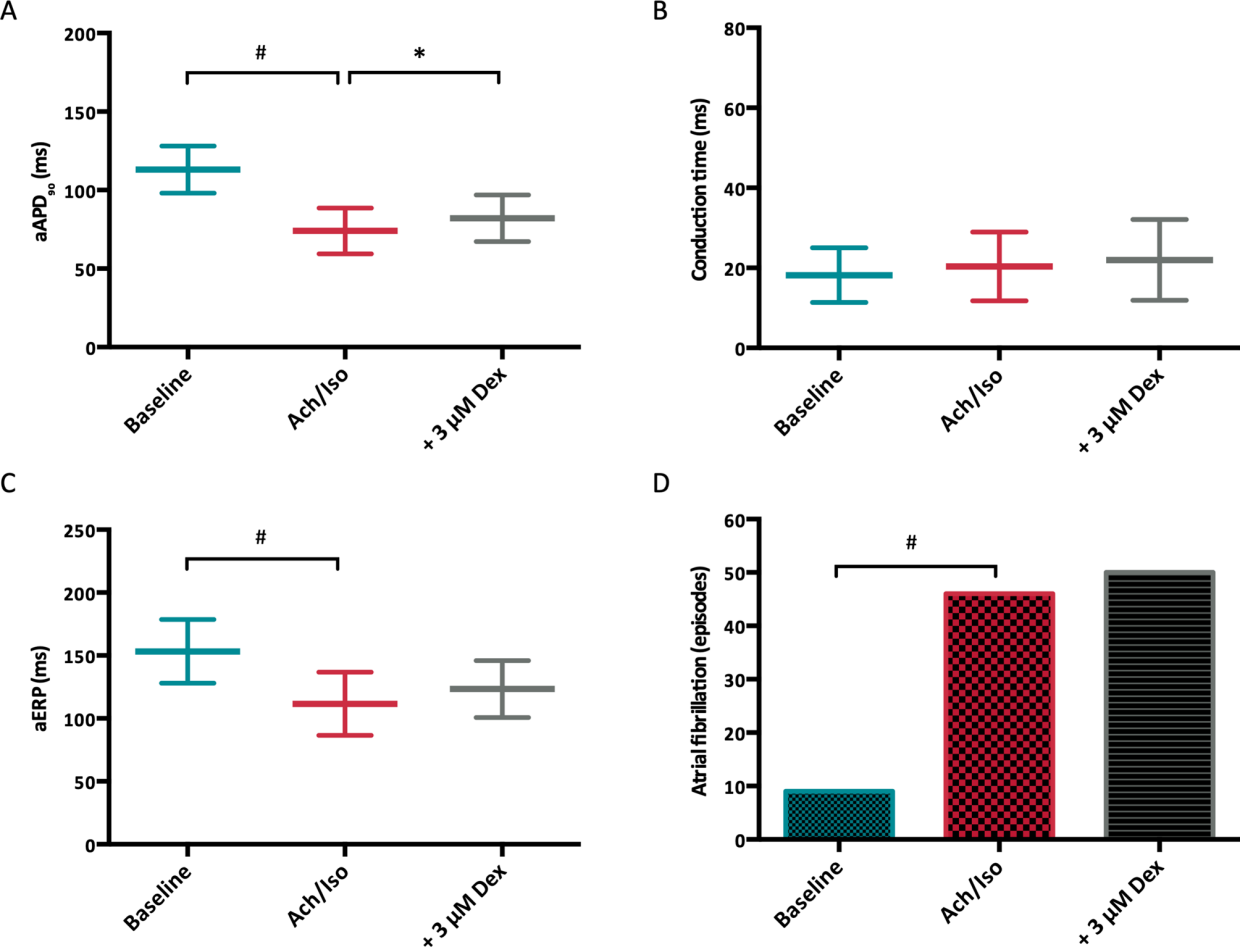
(**A**) Impact of acetylcholine (Ach)/ isoproterenol (Iso) and dexmedetomidine (Dex) on atrial APD_90_ (aAPD_90_). (**B**) Effect of Ach/Iso and dexmedetomidine on atrial conduction time. (**C**) Influence of Ach/Iso and dexmedetomidine on atrial effective refractory periods (aERP). (**D**) Incidence of atrial fibrillation episodes under baseline conditions, with Ach/Iso and with additional dexmedetomidine, respectively (# = p < 0.05 compared to baseline conditions; * = p < 0.05 compared to sole Ach/Iso treatment).

Administration of acetylcholine and isoproterenol significantly reduced atrial APD_90_ from 113 ± 11 ms to 74 ± 5 ms (p < 0.01) and effective refractory periods from 153 ± 25 to 112 ± 25 ms (p < 0.01; Fig. 7). Atrial APD_50_ was reduced by acetylcholine and isoproterenol (from 76 ± 10 to 47 ± 7 ms; p < 0.01) but remained stable after dexmedetomidine treatment (48 ± 9 ms, p = ns). Interatrial conduction time was not altered under the influence of acetylcholine and isoproterenol (20.4 ± 2.3 ms vs. 18.2 ± 2 ms under baseline conditions, p = ns). Additional infusion of dexmedetomidine prolonged atrial APD_90_ (to 82 ± 6 ms, p < 0.01) but did not alter effective refractory periods (to 123 ± 23 ms, p = ns). Atrial conduction time remained unchanged after dexmedetomidine treatment (to 22 ± 3.3 ms, p = ns).

Under baseline conditions, 9 episodes of atrial fibrillation lasting longer than 1 s were inducible. After additional infusion of acetylcholine and isoproterenol, 46 episodes of atrial fibrillation occurred (p < 0.05). Additional dexmedetomidine did not reduce the number of atrial fibrillation episodes (50 episodes, p = ns).

## Discussion

This study demonstrates divergent electrophysiologic effects of dexmedetomidine in different arrhythmia models. No proarrhythmic effects were observed under sole dexmedetomidine administration in the presence of a stable repolarization duration. In a sensitive model of acquired LQT2, torsade de pointes episodes were substantially prevented by dexmedetomidine. No proarrhythmic effects were induced by dexmedetomidine in experimental models of reduced repolarization reserve and atrial fibrillation.

### Dexmedetomidine’s effects on ventricular repolarization

In this study, dexmedetomidine did not significantly alter cardiac repolarization or effective refractory periods. Our results might explain why previous clinical studies found conflicting results of dexmedetomidine’s effects on cardiac repolarization duration^3–7^. Of note, spatial dispersion of repolarization was significantly reduced after dexmedetomidine treatment. This is in line with a previous randomized study that reported on a reduced T_peak_-T_end_ interval after dexmedetomidine treatment^9^.

The distribution of different cell types with different ion channel distribution either within the ventricular wall or between different areas of the ventricles can create a voltage gradient which is reflected by the spatial dispersion of repolarization^18^. For instance, subepicardial M cells possess a larger late sodium current but a reduced potassium current I_Ks_, leading to a longer action potential as compared to epicardial or endocardial cells^20,21^. This results in a transmural dispersion of repolarization which can be reduced by different calcium^22^ or sodium channel inhibitors^23,24^. The reduction of spatial dispersion of repolarization may be explained by different electrophysiological properties of dexmedetomidine: previous studies ascertained that dexmedetomidine inhibits sodium and calcium channels^14–16^. This interplay might explain the reduction of spatial dispersion of repolarization observed in this study, even though this statement remains speculative due to the experimental setup. Since dexmedetomidine inhibits different ion channels, the beneficial effects of dexmedetomidine might be present in most (proarrhythmic) conditions. Similar observations have been made with other antiarrhythmic drugs targeting different ion channels such as ranolazine^25,26^ or amiodarone. Targeting different ion channels might be the best approach for treating arrhythmias since it does not bear the potential proarrhythmic effects observed with different potassium-channel blocking agents (such as sotalol^18^).

An increased spatial dispersion of repolarization is regarded as major arrhythmic mechanism for drug-induced proarrhythmia since it facilitates the occurrence of triggered activity and promotes the perpetuation of arrhythmias^8,27^. In addition, an increased spatial dispersion of repolarization better predicts the occurrence of drug-induced arrhythmias compared to the sole action potential duration^28^. It is worthy of note that a reduction of spatial dispersion is regarded as major antiarrhythmic mechanism in acquired long^19^ and short QT syndrome^29^.

Dexmedetomidine rendered the action potential more rectangular, as indicated by a decrease of the ratio of APD_90_/APD_50_. Rectangulation of the action potentials reduces the time window for re-activation of sodium channels during the vulnerable phase of the action potential and thereby prevents drug-induced arrhythmias^30^. Due to these above-named changes, the ventricular vulnerability as assessed by programmed ventricular stimulation was not increased in the presence of dexmedetomidine. Consistently, no torsade de pointes were observed under hypokalemic conditions in bradycardic hearts after dexmedetomidine administration. These findings support the clinical evidence that dexmedetomidine is not proarrhythmic and can be regarded as safe drug.

### Dexmedetomidine’s effects on acquired long QT syndromes

In this study, erythromycin and veratridine were employed for induction of long QT syndromes type 2 and a model of reduced repolarization reserve, respectively. Both agents are established for the pharmacological simulation of these models^17,24^ and lead to a substantial arrhythmogenicity by prolonging cardiac repolarization duration and amplifying spatial dispersion of repolarization.

In the LQT2-group, dexmedetomidine further prolonged cardiac repolarization and reduced spatial dispersion of repolarization. It remains speculative why dexmedetomidine further prolonged cardiac repolarization in the LQT2-group but did not have a substantial effect on action potential duration and QT interval when administered alone. To explain this conflict, the LQT2 model could be regarded as model of an impaired repolarization reserve. According to this concept of repolarization reserve, drug mediated inhibition of distinct ion channels can be compensated by other ion channels with redundant properties^31^. Following this concept, I_Kr_ inhibition by erythromycin might reveal further ion channel inhibiting properties of dexmedetomidine, as indicated by a further repolarization prolongation in this study. Consistently, dexmedetomidine administration prolonged the QT interval in a child with a high clinical suspicion of congenital long QT syndrome type 2^6^. It is worthy of note that a further prolongation of repolarization duration is not necessarily proarrhythmic but can be even antiarrhythmic under certain circumstances^30^. As indicated above, a reduction of spatial dispersion is a crucial antiarrhythmic mechanism in acquired long QT syndrome^8^ since it reduces the occurrence of triggered activity and impedes the perpetuation of torsade de pointes^8,27^. Thereby, dexmedetomidine substantially suppressed torsade de pointes in erythromycin-pretreated hearts. Our findings further support the results of a previous experimental study that found protective effects of dexmedetomidine in a methoxamine-sensitized rabbit model of acquired long QT syndrome type 2^13^ and demonstrate that the reduction of spatial dispersion seems to be the crucial antiarrhythmic mechanism.

In the veratridine-group, further administration of dexmedetomidine did neither alter cardiac repolarization duration nor spatial dispersion of repolarization. As a consequence, dexmedetomidine did not lead to further arrhythmogenicity in veratridine-pretreated hearts. These divergent electrophysiologic effects of dexmedetomidine in acquired long QT syndrome type 2 and the veratridine group could be most likely explained by direct electrophysiologic alterations of either sodium or potassium channels but cannot be fully elucidated due to the experimental setup. However, previous studies have already indicated direct electrophysiologic effects of dexmedetomidine on potassium^16^, sodium^14,15^ and calcium channels^15,32^.

### Dexmedetomidine’s impact on atrial fibrillation

A combination of acetylcholine and isoproterenol was employed to shorten atrial repolarization duration and thereby induce atrial fibrillation. Even though this pharmacological induction does not fully reflect the complex structural electrophysiologic changes observed in chronic atrial fibrillation, it is an established model to investigate the impact of different drugs on atrial electrophysiology^33,34^. In this study, additional administration of dexmedetomidine prolonged the atrial action potential duration without altering effective refractory periods. The prolongation of atrial action potentials mediated by dexmedetomidine might be explained by the α_2_-adrenoreceptor agonism which might reverse the autonomic stimulation induced by acetylcholine and isoproterenol. Still, no antiarrhythmic properties were observed as dexmedetomidine did not suppress atrial fibrillation in this model. This is in line with a recent large randomized trial that investigated the effect of dexmedetomidine in patients undergoing cardiac surgery and found no protective effect of dexmedetomidine concerning the occurrence of postoperative atrial arrhythmias^35^.

## Limitations

The experiments in this study have been performed employing a whole-heart setup. Therefore, this setup does not allow to measure direct drug effects on different ion channels. However, the whole-heart model employed is one of the most sensitive models when studying cardiac safety. Accordingly, the outstanding role of the rabbit whole-heart model has been outlined in a recent important review on animal models^36^. Underlying reasons for this special role are the comparable configurations of action potentials (both rabbit and human action potentials possess a plateau phase due to similar potassium currents)^18^ and the similar patterns of complex ventricular arrhythmia in human and rabbit hearts^37^. Still, a direct transfer of the findings obtained in this study is not possible.

## Conclusion

In this study, dexmedetomidine did not substantially influence cardiac repolarization duration and significantly reduced spatial dispersion of repolarization in the presence of a preserved repolarization reserve. As a result, no drug-mediated proarrhythmia was observed. Dexmedetomidine exerted antiarrhythmic effects in an experimental model of long QT syndrome type 2 by reducing spatial dispersion of repolarization. Of note, dexmedetomidine treatment was not proarrhythmic in an experimental model of long QT syndrome type 3 and in a whole-heart model of atrial fibrillation. To summarize, our results indicate a safe electrophysiologic profile of dexmedetomidine in different arrhythmia models.

## Data Availability

The datasets generated during and analysed during the current study are available from the corresponding author on reasonable request.

## References

[1] Castillo RL (2019). Dexmedetomidine improves cardiovascular and ventilatory outcomes in critically Ill patients: Basic and clinical approaches. Front. Pharmacol..

[2] Talke P, Richardson CA, Scheinin M, Fisher DMJA (1997). Postoperative pharmacokinetics and sympatholytic effects of dexmedetomidine. Anesth. Analg..

[3] Görges M (2015). Changes in QTc associated with a rapid bolus dose of dexmedetomidine in patients receiving TIVA: a retrospective study. Paediatr. Anaesth..

[4] Kako H, Krishna SG, Sebastian R, Smith K, Tobias JD (2015). Effect of dexmedetomidine on the QT interval in pediatric patients undergoing general anesthesia. J. Anesth..

[5] Görges M (2019). Effects of dexmedetomidine on myocardial repolarization in children undergoing general anesthesia: A randomized controlled trial. Anesth. Analg..

[6] Burns KM, Greene EA (2014). Long QT syndrome unmasked by dexmedetomidine: A case report. Congenit Heart Dis.

[7] Char D (2013). The effects of ketamine on dexmedetomidine-induced electrophysiologic changes in children. Paediatr Anaesth.

[8] Frommeyer G, Eckardt L (2016). Drug-induced proarrhythmia: Risk factors and electrophysiological mechanisms. Nat Rev Cardiol.

[9] Kim NY (2016). Effect of dexmedetomidine on heart rate-corrected QT and Tpeak-Tend intervals during robot-assisted laparoscopic prostatectomy with steep trendelenburg position: A prospective, randomized, double-blinded, Controlled Study. Medicine (Baltimore).

[10] Opthof T (2007). Dispersion of repolarization in canine ventricle and the electrocardiographic T wave: Tp-e interval does not reflect transmural dispersion. Heart Rhythm.

[11] Liu Y (2020). Dexmedetomidine reduces atrial fibrillation after adult cardiac surgery: A meta-analysis of randomized controlled trials. Am. J. Cardiovasc. Drugs.

[12] Yoshitomi O (2012). Direct protective effects of dexmedetomidine against myocardial ischemia-reperfusion injury in anesthetized pigs. Shock.

[13] Tsutsui K (2012). Dexmedetomidine and clonidine inhibit ventricular tachyarrhythmias in a rabbit model of acquired long QT syndrome. Circ. J..

[14] Stoetzer C (2016). Inhibition of the cardiac Na+ channel α-subunit Nav1. 5 by propofol and dexmedetomidine. Naunyn Schmiedebergs Arch. Pharmacol..

[15] Yang L (2021). Dexmedetomidine exhibits antiarrhythmic effects on human-induced pluripotent stem cell-derived cardiomyocytes through a Na/Ca channel-mediated mechanism. Ann. Transl. Med..

[16] Kawano T, Yamazaki F, Chi H, Kawahito S, Eguchi S (2012). Dexmedetomidine directly inhibits vascular ATP-sensitive potassium channels. Life Sci..

[17] Ellermann C (2020). Propofol abolishes torsade de pointes in different models of acquired long QT syndrome. Sci. Rep..

[18] Ellermann C, Wolfes J, Eckardt L, Frommeyer G (2021). Role of the rabbit whole-heart model for electrophysiologic safety pharmacology of non-cardiovascular drugs. Europace.

[19] Ellermann C (2018). Antiarrhythmic effect of antazoline in experimental models of acquired short-and long-QT-syndromes. Europace.

[20] Sicouri S, Antzelevitch C (1991). A subpopulation of cells with unique electrophysiological properties in the deep subepicardium of the canine ventricle The M cell. Circ. Res..

[21] Antzelevitch C (2010). M cells in the human heart. Circ. Res..

[22] Milberg P (2012). Blockade of ICa suppresses early afterdepolarizations and reduces transmural dispersion of repolarization in a whole heart model of chronic heart failure. Br. J. Pharmacol..

[23] Osadchii OE (2012). Impact of Na+ channel blockers on transmural dispersion of refractoriness and arrhythmic susceptibility in guinea-pig left ventricle. Eur. J. Pharmacol..

[24] Frommeyer G (2017). Broad antiarrhythmic effect of mexiletine in different arrhythmia models. Europace.

[25] Frommeyer G (2012). Effect of ranolazine on ventricular repolarization in class III antiarrhythmic drug-treated rabbits. Heart Rhythm.

[26] Frommeyer G (2012). New insights into the beneficial electrophysiologic profile of ranolazine in heart failure: prevention of ventricular fibrillation with increased postrepolarization refractoriness and without drug-induced proarrhythmia. J. Card Fail..

[27] Bossu A (2018). Selective late sodium current inhibitor GS-458967 suppresses Torsades de Pointes by mostly affecting perpetuation but not initiation of the arrhythmia. Br. J. Pharmacol..

[28] Ellermann C (2020). Proarrhythmic effect of acetylcholine-esterase inhibitors used in the treatment of Alzheimer’s disease: Benefit of rivastigmine in an experimental whole-heart model. Cardiovasc. Toxicol..

[29] Milberg P (2007). Reduction of dispersion of repolarization and prolongation of postrepolarization refractoriness explain the antiarrhythmic effects of quinidine in a model of short QT syndrome. J. Cardiovasc. Electrophysiol..

[30] Hondeghem L, Carlsson L, Duker G (2001). Instability and triangulation of the action potential predict serious proarrhythmia, but action potential duration prolongation is antiarrhythmic. Circulation.

[31] Roden DM (2008). Repolarization reserve: A moving target. Circulation.

[32] Zhao J, Zhou C-L, Xia Z-Y, Wang L (2013). Effects of dexmedetomidine on L-type calcium current in rat ventricular myocytes. Acta Cardiol. Sin.

[33] Frommeyer G (2017). Effective suppression of atrial fibrillation by ivabradine: Novel target for an established drug?. Int. J. Cardiol..

[34] Schüttler D (2020). Animal models of atrial fibrillation. Circ. Res..

[35] Turan A (2020). Dexmedetomidine for reduction of atrial fibrillation and delirium after cardiac surgery (DECADE): A randomised placebo-controlled trial. Lancet.

[36] Clauss S (2019). Animal models of arrhythmia: Classic electrophysiology to genetically modified large animals. Nat. Rev. Cardiol..

[37] Panfilov AV (2006). Is heart size a factor in ventricular fibrillation? Or how close are rabbit and human hearts?. Heart Rhythm.

